# Tumor-derived Exosome Promotes Metastasis via Altering its Phenotype and Inclusions

**DOI:** 10.7150/jca.48043

**Published:** 2021-05-19

**Authors:** Yu Zhou, Fan Chen, Xiaodong Xie, Huifang Nie, Shu Lian, Chunlian Zhong, Chengbin Fu, Weiyu Shen, Bifei Li, Yongqing Ye, Yusheng Lu, Lee Jia

**Affiliations:** 1 Institute of Oceanography, Minjiang University, Fuzhou, 350108, China.; 2 Cancer Metastasis Alert and Prevention Center, and Biopharmaceutical Photocatalysis of State Key Laboratory of Photocatalysis on Energy and Environment, College of Chemistry; Fujian Provincial Key Laboratory of Cancer Metastasis Chemoprevention and Chemotherapy, Fuzhou University, Fuzhou, 350116, China.; 3 Department of Breast Surgery, Fujian Medical University Union Hospital, Fuzhou 350001, China.; 4 Fujian Sanyi Hematopoietic Technology Co. Ltd., Fuzhou 350108, China.

**Keywords:** Tumor-derived exosome, miRNA, α-SMA, early tumor, advanced tumor, cancer metastasis

## Abstract

Although tumor-derived exosomes play an important role in the process of metastasis, differences in exosomes secreted by the same cells at different stages or conditions have not been noticed by most of the relevant researchers. Here we developed a lung cancer model in nude mice, and the phenotype and inclusions of exosomes secreted by early and advanced tumors were analysed. The size distribution and surface topography of these two exosomes were not significantly different, but the expression of CD63 in early tumor exosome (E-exosome) was significantly lower than that in advanced tumor exosome (A-exosome). α-SMA expression on HELF cells treated with A-exosome was significantly higher than that treated with E-exosome. The ability of A-exosome to promote the migration of A549 cells was better than E-exosome. Furthermore, small RNA sequence showed that only 3 of the 171 detected-small RNAs were expressed simultaneously in both exosomes. These findings proved that there are significant differences in inclusions and functions between the early and late exosomes of the same tumor. The study highlights the importance of exosomes in cancer metastasis, and might suggest exosomes can be used as biomarkers and therapeutic targets for cancer metastasis.

## Introduction

Exosomes are nanosized (30-200 nm) vesicles that secreted by almost all cells 1, 2. The surface of exosomes is rich of tetraspanins (CD9, CD63, CD81 and CD82) and immune regulator molecules (MHCI, MHCII, and CD86) as well as some lipid rafts. In many recent researches, tetraspanins are wildly used in exosomes recognition 3-7. In addition, with the difference of original cell type, the surface of exosome often contains some specific biomarkers which are abundant on original cell surface 8, 9. Furthermore, the inside of the exosomes is also rich in enzymes (GAPDH, Enolase, PK, ATPase, PGK1), RNA (miRNA, mRNA), MVB biogenesis (Alix, TSG101, Clathrin, Ubigutin), heat shock proteins (HSP60, HSP70, HSP90, HSC70), signal transduction (EGFR, CDC42, PI3K, ARF1, G proteins, MUC1, B catenin) which come from initial cells 8-10.

Many recent studies have showed that exosomes play an important role in the communication between cells 11-14. It is also demonstrated that miRNAs packaged in exosomes are related to MPC-mediated regulation of muscle fibrogenic cell collagen production 15. Resulting from the small size and good biocompatibility, exosomes have been wildly used as vehicles for miRNA or drug delivery 6, 10, 16, 17. It has also reported that viral resistance can be transferred from activated cells to non-activated cells via exosomes 4. Moreover, exosomes lead to tumor metastasis through inducing vascular leakiness, inflammation, bone marrow progenitor cell recruitment 18. And the integrins on exosomes cause organ-specific tumor metastasis 19-23.

Exosome is a hot research topic, and many related studies have been published 6, 13, 19, 24, 25. However, almost all of those researches have neglected the difference between the exosomes from same cells at different stages or conditions, especially for the cases that using exosomal miRNA for cancer diagnostics and therapy 26. We hypothesized that exosomes are tools by which cells communicate with outside 1, 13, 14 to fulfill the needs of its own function 27-30. Hence, the exosomes secreted by early and advanced tumors may be different caused by their different needs. In the present study, for the first time, we compared the difference between exosomes secreted by the tumor cell at different stages of metastasis. And found that there was no significant difference in physicochemical properties between them, while the biological phenotype and inclusions (such as miRNAs) having significant differences. Furthermore, the exosomes from differential stage tumors trigger functional differences in promoting tumor metastasis.

## Materials and Methods

Cell culture, Extraction and characterization of exosomes, small RNA high-throughput sequencing and data processing, miRNA functional analysis, effects of exosome on cancer metastasis, and statistical analysis are described in the **Supplementary Information**.

## Results and Discussion

### Characterization of Physicochemical Properties of Exosomes

**Figure 1:**
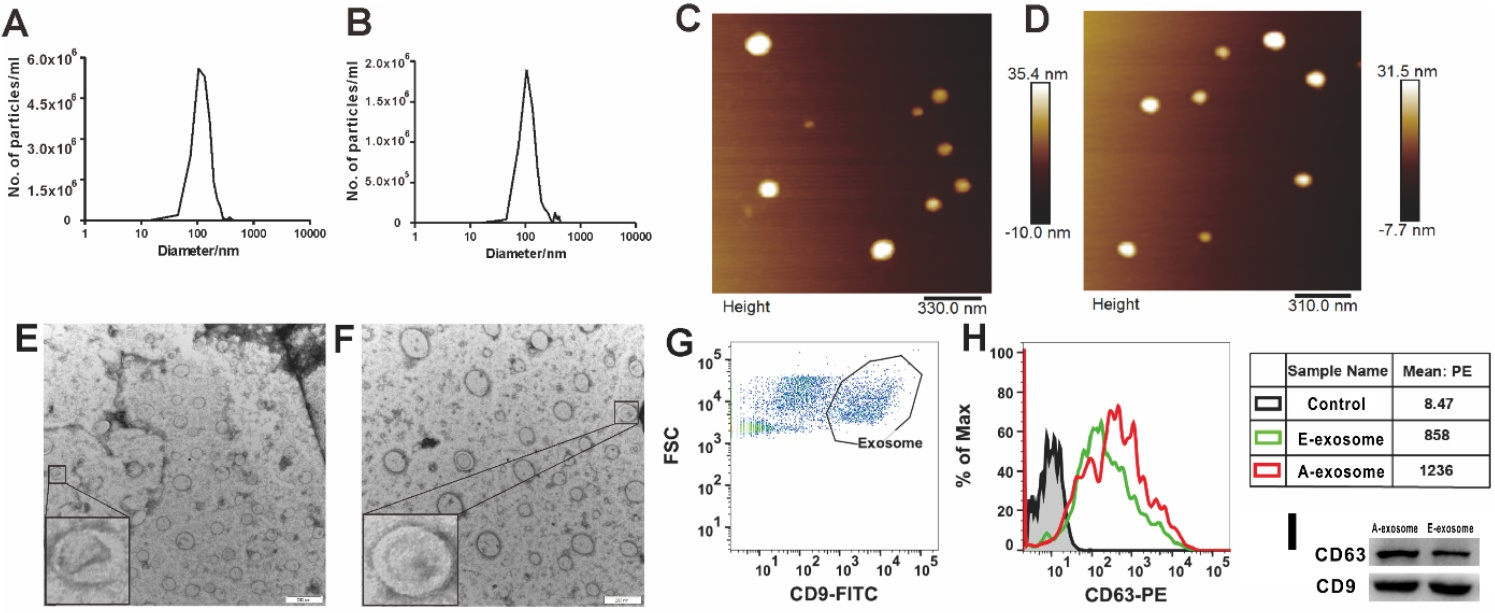
** Characterization of exosomes. A and B,** Nanoparticle Tracking Analysis (NTA) show the size distribution and concentration of E-exosome (**A**) and A-exosome (**B**). **C and D**, atomic force microscopy (AFM) reveal the size and morphology of E-exosome(**C**) and A-exosome (**D**). **E and F**, morphological images of E-exosome (**E**) and A-exosome (**F**) with transmission electron microscopy (TEM). Scale bars, 200 nm. **G and H,** flow cytometry plots of exosomes that excluded the unassigned events and artifacts and identified the cell population as exosomes characterized by CD9+ (Exosome gate, **G**), the histograms are representatives of CD63 expression from E-exosome and A-exosome (the right tables, **H**). **I,** western blot analysis of CD63 and CD9 expression in E-exosome and A-exosome.

The early and advanced stages are determined according to the size of the tumor and the time of tumor implantation 31. In the present study, we defined the mice intrapulmonary implanted with A549 cells for 14 days (tumor diameter < 2 mm) as early stages, and 35 days (tumor diameter > 5 mm) as advanced stages. To investigate whether there are differences between exosomes secreted by early tumors (E-exosome) and advanced tumors (A-exosome), we first examined the physical and chemical properties of exosomes. The size distributions of two kinds of exosomes were determined with Nanoparticle Tracking Analysis (NTA, Nanosight), the diameters of two kinds of exosomes are range from 45 to 200 nm (**Fig. 1A-B**). Atomic force microscope imaging (AFM, Bruker Co, Germany, **Fig. 1C-D**) and transmission electron microscopy (TEM, JEM-1400plus, Japan, **Fig. 1E-F**) results proved the good homogeneity of E-exosome and A-exosome. The Flow cytometer (Becton Dickinson FACS AriaIII cell sorter) was used to determine the expression of CD9 and CD63 on the exosomes derived from early tumor and advanced tumors. The expression of CD63 were significant differences between CD9+ E-exosome and CD9+ A-exosome. Compared with CD9+ E-exosome, the expression of CD63 on CD9+ A-exosome obviously increased (**Fig. 1G-H**). To further confirm the expression of CD9 and CD63 observed by flow cytometry, western blot analysis was performed. The result was consistent with that obtained from the flow cytometric analysis (**Fig. 1I**). Overall, there was no significant difference in the size and shape of the two exosomes, but the biological phenotypes were significantly different.

### A-exosome promotes cancer metastasis

**Figure 2:**
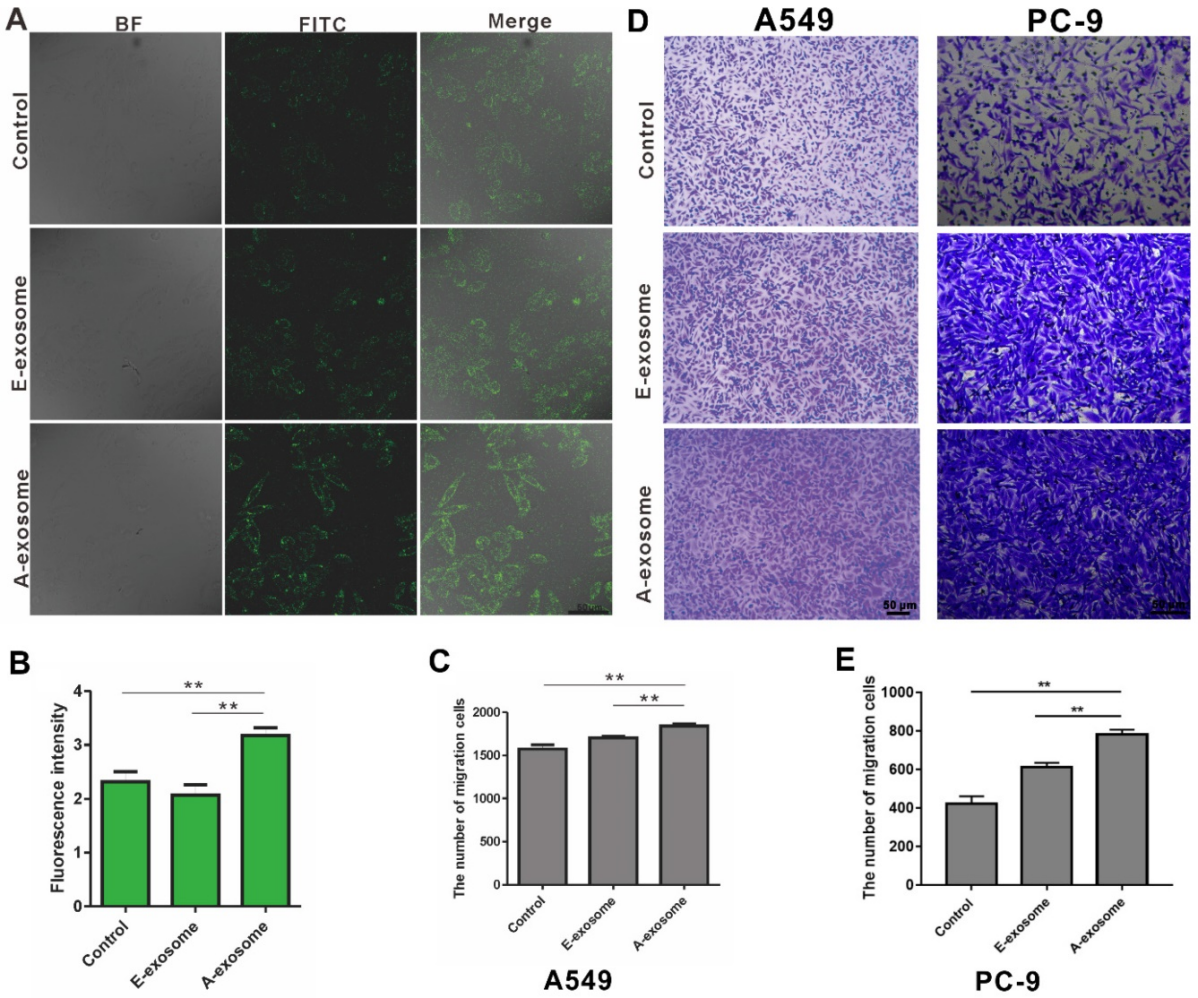
** The effects of E-exosome and A-exosome in promoting tumor metastasis. A,** The expression of α-SMA on HELF cells after treated with 1x10^9^ total E-exosome and A-exosome. **B,** Quantitative analysis of α-SMA expression by fluorescence intensity. **C-E,** Representative photos and quantification of exosomes promoting the migration of A549 and PC-9 cells through transwell model. Bars represent the mean ± SD (n=3); **, P < 0.01.

The effects of tumor-derived exosomes to distance organ have been widely reported, and these effects may promote cancer metastasis 32-34. To determine the effects of E-exosome and A-exosome on metastasis, two kinds of exosomes were incubated with human embryonic lung fibroblast (HELF) cells, and the expressions of α-SMA on HELF cells were examined after incubation. The expression of α-SMA on HELF cells after treated with A-exosome is obviously high than that after treated with E-exosome (**Fig. 2A-B**). The expression of α-SMA indicates the transformation of fibroblast to myofibroblast, and the fibroblast-myofibroblast transition can support tumor growth, vascularization, and metastasis 35. These results proved that A-exosome promotes cancer metastasis by inducing the transformation of fibroblast to myofibroblast. The proliferation of myofibroblasts increased lymph angiogenesis, and it also associated with lymph node metastasis. While E-exosome-treated HELF cells did not exhibit this level of fibroblast-myofibroblast transition. To further examine the function of two exosomes to cancer metastasis, the effects of two kinds of exosomes to A549 cells were determined. After treating with E-exosome and A-exosome respectively, the migration of A549 were determined. The migration of A549 cells from upper chamber to lower chamber of transwell were rapidly increased after incubation with A-exosome, and the effect of E-exosome in promoting migration were obviously less than that of A-exosome (**Fig. 2C-D**). Moreover, the migration of PC-9 cells after treatment with exosome was consistent with A549 cells (**Fig. 2D-E**). This migration-promoting effect can be considered as an active regulation of tumor tissue to cancer metastasis through exosomes. Exosomes are the tools that cells used to communicate with outside and perform functions. In the advanced stage of tumor, the tumor has the characteristics of metastasis, and the exosome secreted by advanced tumor can contribute to tumor metastasis. There is no tendency to metastases in the early stage tumor, therefore the exosome secreted by the same tumor in early stage has no such functions of promoting metastasis.

### The expression of miRNA on E-exosome and A-exosome is significantly different

The process of miRNA analysis is shown in**Fig. S1**. We first removed the sequences of low quality, the sequence of the unpredictable base sequence proportion over 10%, the sequence with 5' or 3' broken and poly A/T/G/C sequence. The filtered sequences are clean reads. As shown in **Table S1**, there are 91.31% clean reads in E-exosome and 89.32% clean reads in A-exosome. The frequency percentage of sequence length between 26-30 nt were significantly increased in A-exosome than in E-exosome (**Fig. S2A-B**). After the length filtering, all the reads were mapped to the reference sequence. The percentage of mapped small RNA, mapped forward sequence RNA and mapped reverse sequence RNA were showed in **Table S2**. There are more mapped sRNAs in A-exosome (18.13%) than that in E-exosome (14.38%). The density of reads of all chromosomes in each sample was compared to that of the genome. The results of distribution of reads on each chromosome were examined by Circos mapping. The different distribution of reads on each chromosome between E-exosome and A-exosome proved that the inner reads in two kinds of exosomes are different (**Fig. S2C-D**). Overall, the amount and type of reads in E-exosome and A-exosome are different, which will lead to functional differences between the exosomes.

**Figure 3:**
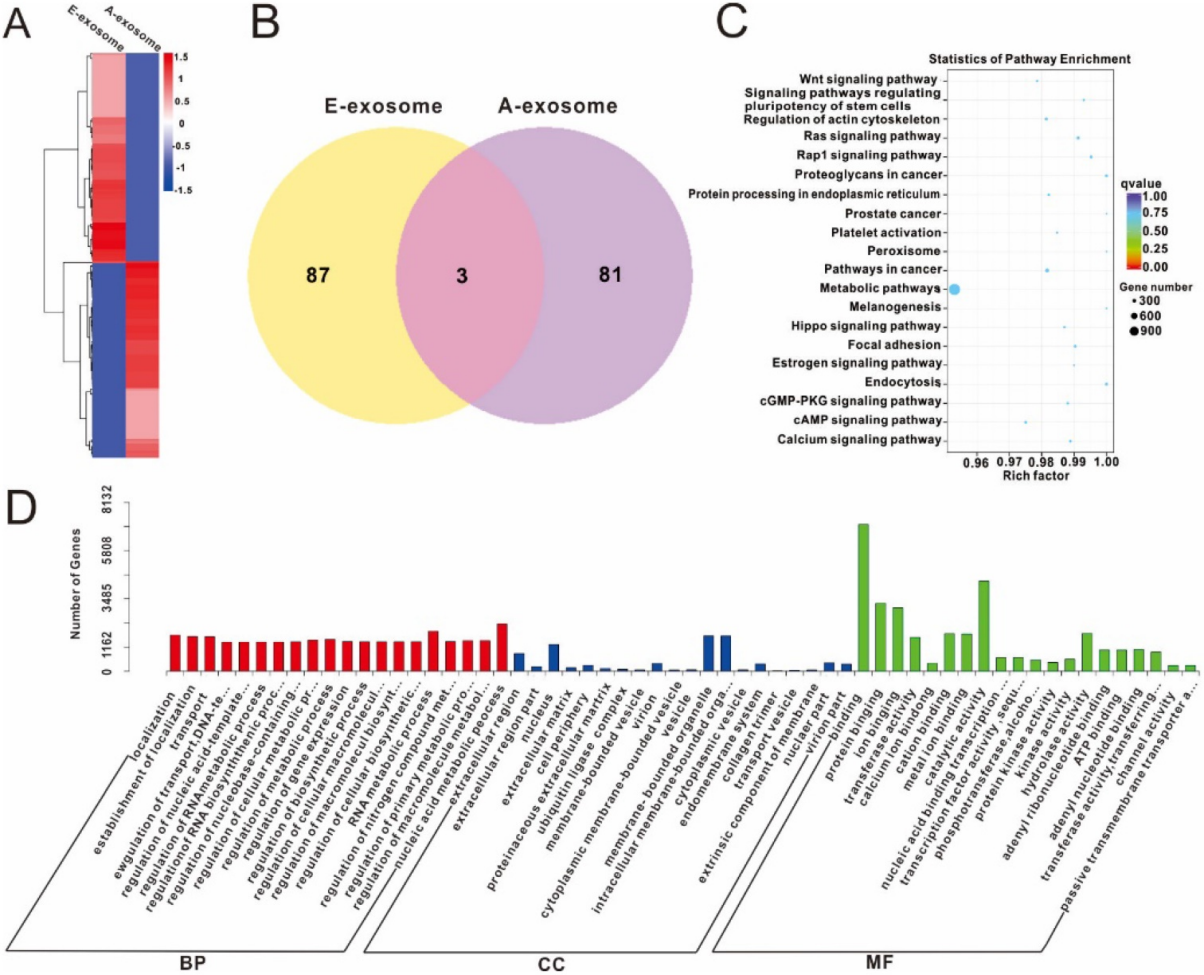
** miRNA Prediction and Differential Expression Analysis. A,** Heatmap diagram of differential miRNA expression between E-exosome and A-exosome. **B,** overlap graph of differential miRNA expression in each exosome. **C,** Gene Ontology enriched histogram of target genes (E-exosome vs A-exosome). **D,** KEGG enrichment point map of target genes (E-exosome vs A-exosome).

Furthermore, the differential expression of miRNA on E-exosome and A-exosome were analyzed by heat map. And we observed the significant changes in two kinds of exosomes (**Fig. 3A**). The result was also expressed in Venn graph. The entire database includes 171 unique miRNAs, while only 3 miRNAs were measured in one platform. Eighty-seven miRNAs were measured in E-exosome versus 81 miRNAs were measured in A-exosome (**Fig. 3B,** and the details were shown in **Table S3**). There results indicated that expression of miRNA on E-exosome and A-exosome is significantly different. The unannotated miRNA and annotated miRNA sequences were also analyzed, and the sequences were provided in **Table S4**.

The target genes of miRNA were predicted by miRanda, and the results were showed in **Table S5**. The abundant of miR1260b in A-exosome will target to specific mRNA and gene which will cause difference in its function. The relative functions of the target gene were determined by Gene Ontology, and the results (E-exosome vs A-exosome) were illustrated in **Fig. 3C**. These differences in miRNA between E-exosome and A-exosome indicating these two exosomes have different functions. In living organisms, different genes coordinate with each other to exercise their biological functions, and Pathway's significant enrichment can determine the most important biochemical metabolic pathways and signal transduction pathways involved in candidate target genes. Kyoto Encyclopedia of Genes and Genomes (KEGG) is the main public database about Pathway 36. The Statistics of Pathway Enrichment of KEGG was shown in **Fig. 3D** (E-exosome vs A-exosome). These results suggested that the differential expression of miRNAs on E-exosome and A-exosome are related to Wnt signaling pathway, signaling pathways regulating pluripotency of stem cells, RAS signaling pathway, proteoglycans in cancer, prostate, pathways in cancer, metabolic pathways, melanogenesis, focal adhesion, endocytosis, c-GMP-PKG signaling pathway, c-AMP signaling pathway and calcium signaling pathway. The present study indicated that the E-exosome and A-exosome regulate different pathways by containing different miRNAs.

**Figure 4:**
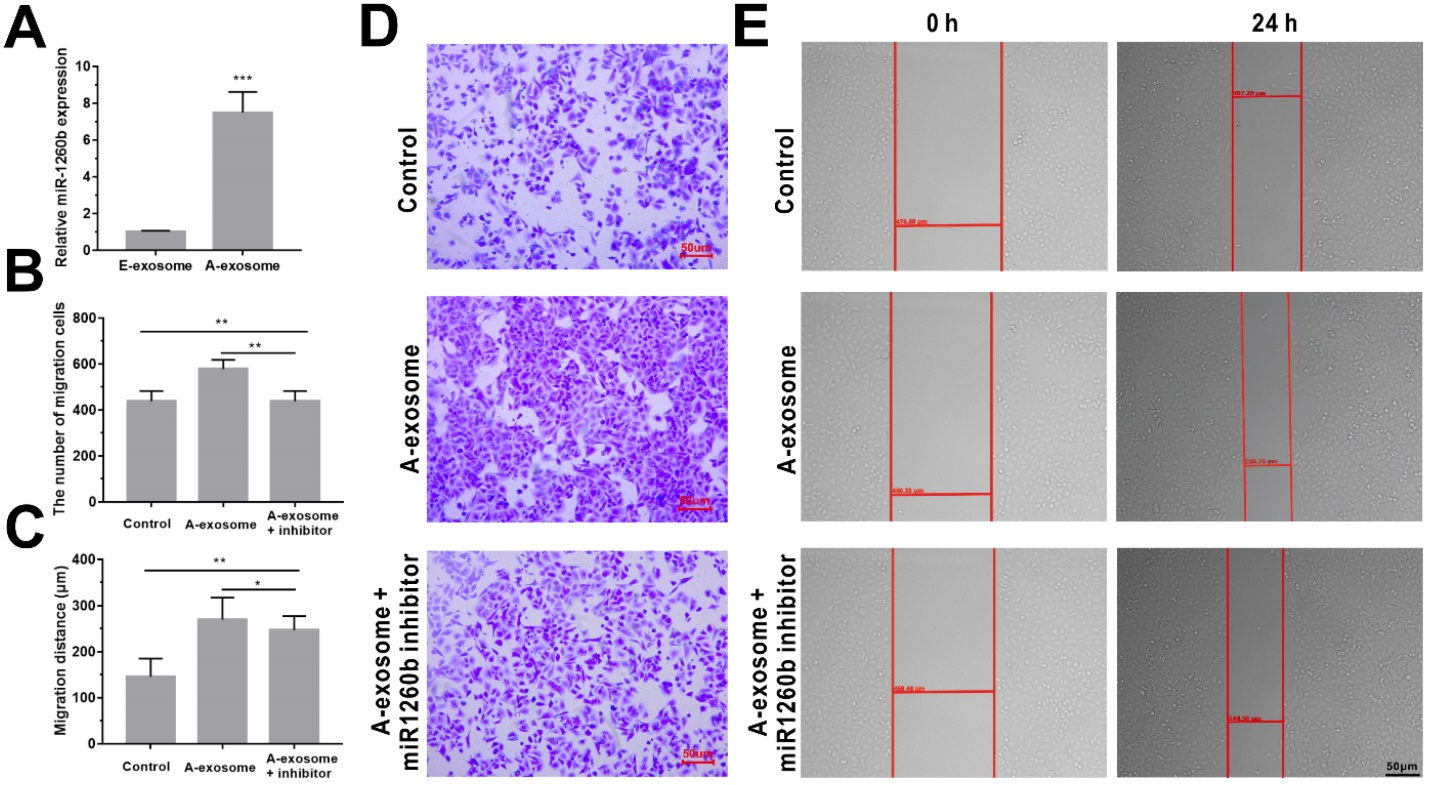
** Effect of hsa-miR1260b in A-exosome. A,** relative hsa-miR1260b expression in A-exosome and E-exosome. **B and C,** quantitative analysis of the invasion (**B**) and migration (**C**) ability of A549 cells by transfection of hsa-miR1260b inhibitor and treatment of A-exosome. **D and E,** representative images showing that the invasion (**D**) and migration (**E**) ability of the A549 cells were processed by hsa-miR1260b inhibitor and treatment of A-exosome. Bars represent the mean ± SD (n=3); *, P < 0.05; **, P < 0.01; ***, P < 0.001.

Among these detected sequences, hsa-miR1260b is significantly expressed in A-exosome but not expressed in E-exosome (**Table S3**). Hsa-miR1260b expression levels in exosomes (A-exosome and E-exosome) were validated by real-time PCR. As shown in **Fig. 4A**, hsa-miR1260b expression was significantly higher in A-exosome compared with the E-exosome. Interestingly, hsa-miR1260b is reported to be related to promotion of tumor migration and invasion 37, 38. We hypothesized that A-exosome carrying a large amount of hsa-miR1260b may cause the promoting effect to the cancer metastasis in distance tissues. To further confirm the effect of hsa-miR1260b in A-exosome. Transfection of hsa-miR1260b inhibitor into A549 cells for 24 h, followed by incubation with A-exosome for 24 h. Then cell transwell and wound healing assays were performed. We observed significantly decreased A549 cells invasion and migration in hsa-miR1260b inhibitor transfected cells compared with A-exosome treated cells (**Fig. 4B-E**). The result indicated that the ability of A-exosome to promote the invasion and migration of A549 cells was better than E-exosome may be caused by the overexpression of hsa-miR1260b.

**Figure 5:**
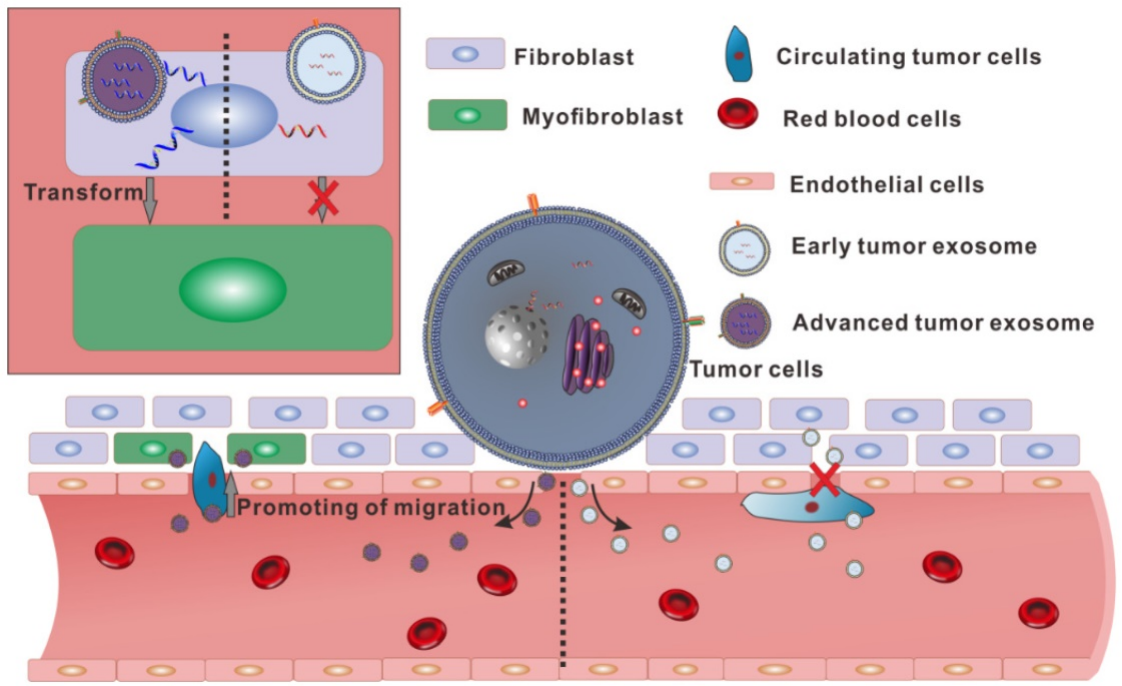
Schematic diagram showing the different functions of the two exosomes secreted by homologous cells.

To sum up, there is no significant difference in appearance between the exosomes secreted by the same tumor in different stages. However, in the content of the two exosomes, there are huge differences in small RNA. Especially, the high expression of hsa-miR1260b in A-exosome may relate to the increasing of migration of A549. And over-expression of α-SMA in fibroblast after treated with A-exosome also contributed to lymph angiogenesis and lymph node metastasis (**Fig. 5**). These differences make the two kinds of exosomes from the same tumor have different functions in promoting transformation of distal tissue and enhancing the migration of tumor cells. These results showed that the same cells could secrete various exosomes with different functions.

In conclusion, the present studies demonstrated that tumor-derived exosome promotes metastasis via altering its phenotype and inclusions. This new discovery might suggest exosomes can be used as biomarkers for cancer metastasis. Furthermore, these new findings also provide further insights for optimized cancer metastatic prevention strategies such as by targeting and eliminating blood oncogenic exosomes.

## Supplementary Material

Supplementary materials and tables.
