# Supplementary material for: Tumor-derived Exosome Promotes Metastasis via Altering its Phenotype and Inclusions
Source: J Cancer. 2021 May 19;12(14):4240–6. doi: 10.7150/jca.48043 (PMC8176431; doi:10.7150/jca.48043)
Supplement: Supplementary file 1 — Supplementary materials and tables. [file jcav12p4240s1.pdf]

Tumor-derived Exosome Promotes Metastasis via Altering its Phenotype and Inclusions

Running Title: Tumor-derived Exosomes are Evolutionary

Yu Zhou[1, 2], Fan Chen[2], Xiaodong Xie[1, 2], Huifang Nie[2], Shu Lian[1, 2], Chunlian Zhong[1], Chengbin Fu[3], Weiyu Shen[2], Bifei Li[2], Yongqing Ye[4], Yusheng Lu[1, 2, *], Lee Jia[1, 2, *]

[1] Institute of Oceanography, Minjiang University, Fuzhou, 350108, China.

[2] Cancer Metastasis Alert and Prevention Center, and Biopharmaceutical Photocatalysis of State Key Laboratory of Photocatalysis on Energy and Environment, College of Chemistry; Fujian Provincial Key Laboratory of Cancer Metastasis Chemoprevention and Chemotherapy, Fuzhou University, Fuzhou, 350116, China.

[3] Department of Breast Surgery, Fujian Medical University Union Hospital, Fuzhou 350001, China.

[4] Fujian Sanyi Hematopoietic Technology Co. Ltd., Fuzhou 350108, China.

*Corresponding authors: Yusheng Lu, Marine Drug R&D Center, Institute of Oceanography, Minjiang University, Xiyuangong Road, University Town, Fuzhou 350108, Fujian, China. Email: lu_yu_sheng@126.com; Lee Jia, Marine Drug R&D Center, Institute of Oceanography, Minjiang University, Xiyuangong Road, University Town, Fuzhou 350108, Fujian, China. Email: cmapcjia1234@163.com.

Supplementary Information

Experimental procedures

Cell culture

Human embryonic lung fibroblast (HELF) cells were cultured in DMEM (high glucose) medium. A549 and PC-9 cells were cultured in RPMI 1640 medium. All the medium were contain 10% exosome-depleted FBS and 1% penicillin-streptomycin. Cells lines were from the Cell Bank of Type Culture Collection of Chinese Academy of Sciences (Shanghai, China).

Extraction of exosomes

Female BALB/C nude mice (four to six weeks old) were purchased from Jiesijie Animal center (Shanghai, China). All animal experiments were approved by our Animal Care and Use Committee to reduce the suffering and using of animals. No statistical method was used to pre-determine sample size. To establish an in situ lung cancer model, the mice were intrapulmonary implanted with A549 cells (1×10^6 cells/mice). The tumor-bearing mice were randomly divided into 2 groups (n=6). Group 1 was executed 14 days later after the injection, and group 2 was executed 35 days later after injection. The tumor tissues (500mg-1000mg/mouse) were carefully collected to avoid containing normal tissue, and tumor-derived exosomes were extracted similarly as described [1]. Briefly, tumor tissue was sliced into 1×1 mm pieces, and the pieces were placed into 10 ml 75 U/ml of collagenase type III. The tissue was then incubated in water bath at 37°C for 20 min. During the

incubation, the tube was shaken slowly. After that, the supernatant was collected and centrifuged at 200 g for 10 min, followed by 1,500 g for 10 min to remove debris. The collected medium was then ultracentrifuged at 100,000g for 2h at 4°C. The exosomes was washed with PBS, followed by another ultracentrifugation at for 2h at 4°C. Exosomes was collected for further characterization and experiments.

Characterization of exosomes

The size distribution of two exosomes samples were determined by nanoparticle tracking analysis (NTA; ZetaView, Germany). Samples were diluted in PBS to achieve a concentration between $1x10^{8}$-$2x10^{9}$/mL, and the results were measured with NTA. The surface appearance of two exosomes samples were measured by Multi Mode 8 Atomic Force Microscope (AFM; Bruker Co., Germany) and Transmission Electron Microscopy (TEM; JEM-1400plus, Japan). For AFM assay, 10 μL samples were added onto freshly cleaved mica and imaging was obtained by using AFM. To obtain the TEM images, 5-10 μL of E-exosome and A-exosome were dropped onto carbon-coated formvar Cu grids. Grids were washed with dd H2o and stained with uranyl acetate. The images were obtained with TEM. The expressions of the biomarkers (CD9 and CD63) were determined by flow cytometry and western blot.

Flow cytometry

E-exosome and A-exosome 30 μl ($1.4x10^{7}$ exosomes) were firstly incubated with 10 μL CD9 Flow Detection beads for 15 min at 25 °C, and then the mixture was diluted to 1 mL and

sharked for 30 min at 25 °C. After that, PBS (containing 100 mM glycine and 2% BSA) were used to stop the reaction. The mixture was centrifuged at 15,000 g for 5 min to remove the un-conjugated exosomes. After washing with PBS and blocking with 10% BSA, the result products were incubated with anti-CD9-FITC antibody (3 μl, Abcam) and anti-CD63-PE antibody (3 μl, Abcam) at 4°C for 1h, respectively. After the incubation, the products were centrifuged at 15,000 g for 5 min and washed with PBS. Background staining was performed by staining exosomes with isotype-matched control. Flow cytometric analysis was carried out on the BD FACSAriaIII (BD Biosciences), and the obtained data were analyzed with FlowJo software.

Western blot analysis

Western blot analysis was used to evaluate the expression of exosome surface markers CD9 and CD63, and performed as previously described protocols [2, 3]. Briefly, exosomes were lysed with RIPA lysis buffer (Thermo Fisher). The protein concentration was determined by using the BCA protein assay kit (Thermo Fisher). The equal qualities of protein were separated by SDS-PAGE (Bio-Rad), and transferred to PVDF membrane (Bio-Rad). The membranes were blocked in 5% skim milk for 1 h at room temperature and then incubated with primary antibodies overnight at 4 ℃. After washing, the membranes were incubated with the following secondary anti-bodies for 1h at room temperature. Chemiluminescent signals were generated by using a Super Signal West Pico Chemiluminescent Substrate kit (Pierce), and detected by using the ChemiDoc XPS system (Bio-Rad).

Small RNA High-throughput sequencing and data processing

Total RNA was extracted with TRIzol Reagent (Life Technologies) and quantified by using a Nanodrop and Qubit assay. Agilent 2100 Bioanalyzer was used to verify the integrity of RNA. Microarray experiments were performed at Beijing Weihui Biological Technology Co., Ltd. China. cDNA library was established with NEXTflex Small RNA-Seq Kit (Bioo Scientific). The raw sequencing reads were processed, followed by read extraction using high-throughput sequencing data analysis. The low quality reads (including the sequence of the unpredictable base sequence proportion over 10%, the sequence with 5' or 3' broken and poly A/T/G/C sequence) were abandoned for the accuracy of the results. The reads after screening are clean reads, and the final clean reads number and its proportion to total raw reads number were obtained. Demultiplexed RNA sequencing data were mapped against our curated human reference transcriptome to obtain miRNA raw read and frequency profiles. The mapped sRNA which can be matched to chromosomes in the genome were counted, and using Circos to map the distribution of reads on each chromosome. The mapped sRNAs were compared with the miRBase to obtain the sequence, secondary structure, length and frequency of matched miRNAs.

The ncRNA was also analysised by comparing the abundance of fragments of other RNA classes, such as tRNAs, snRNAs, snoRNAs, and rRNAs. The repeat classification was performed to avoid repeat reads. The sRNAs were compared to exons and introns of mRNA, and sRNA from mRNA degradation fragments was identified. New miRNA prediction and annotation were performed with miREvo [4] and miRDeep2 [5]. The iconic hairpin structure of the miRNA precursor was used to predict the novel miRNA in sRNA sequences.

Differential expression analysis of miRNA

Raw sequencing reads of predicted miRNAs were used for differential expression analysis. MicroRNA may undergo partial editing of bases, leading to changes in the sequence of seeds, resulting in altered target genes. By comparing the sRNA with the detected sequences of the known, new miRNA mature sequence and their precursors, the miRNA that may have been edited by the base mutation was founded. Statistics on the amount of expression of known and new miRNA in each sample were performed and normalized the expression quality with TPM. A miRNA expression difference analysis was conducted to detect miRNAs with significant differences in expression between E-exosome and A-exosome.

Prediction of miRNA targets

The target genes prediction for known miRNAs and novel miRNAs were conducted with miRanda software, and the relationship between miRNA and target genes was obtained.

Functional analysis of miRNA targets

Gene ontology (GO, http://www.geneontology.org/) term distribution and enrichment analysis were conducted, and enriched Kyoto Encyclopedia of Genes and Genomes (KEGG) pathways were identified [6].

MiR-1260b inhibitor transfection

MiR-1260b inhibitor (Ambion) was transiently transfected into A549 cells with the siPORT

NeoFX transfection agent (Thermo Fisher) according to the manufacturer's instructions. Transfection of hsa-miR1260b inhibitor into A549 cells for 24 h, followed by incubation with A-exosome for 24 h. Then cell transwell and wound healing assays were performed.

Quantitative real-time PCR analysis

Total miRNA from exosomes were extracted using TRIzol Reagent (Life Technologies). These procedures were carried out as described previously [7, 8]. Quantitative real-time RT-PCR was performed in triplicate with an CFX96™ Real-Time PCR Detection System using TaqMan universal PCR master mix according to the manufacture's protocol (Bio-Rad). The TaqMan probes and primers were purchased from Thermo Fisher Scientific. RNU48 was used as endogenous control. The miR-1260b expression levels were determined using the 2-ΔΔCt method.

Effects of E-exosome and A-exosome on cancer metastasis

To determine the different effects of E-exosome and A-exosome on cancer metastasis, the expression of α-SMA on HELF cells after treated with E-exosome and A-exosome were measured by the immunofluorescence assay. Briefly, HELF cells were planted on confocal dishes and cultured at 37 °C for 24h. Then $1x10^9$ total E-exosome or A-exosome were added to the dishes and incubated at 37 °C for another 24h. The cells were washed with PBS (pH 7.4), and then treated with polyoxymethylene for 5 min. The cells were permeabilized with 0.1% Triton X-100 for 5 min, and blocked with 5% BSA for 30 min. The cells were incubated with FITC-labeled anti-α-SMA (Abcam, ab8211; 1:100) at room

temperature for 90 min. After washing with PBS for 5 times, the cells were imaged with confocal fluorescence microscopy (Leica TCS SP8, Germany).

Transwell assay

We also determined the effects of exosomes in promoting cancer cell metastasis. A549 and PC-9 cells (1 x10^5 per well) were added into the upper chamber of transwell and medium contain 20% FBS were added into the lower chamber. After incubation with E-exosome and A-exosome (2 x10^8 per well) at 37 °C for 24 h, the migration cells were fixed with methanol and stained with crystal violet. Each sample was captured with 10 random visual fields with Carl Zeiss Primo Star microscopy (Carl Zeiss, Germany).

Wound healing assay

The migration ability of A549 cells was assessed in a classical wound healing assay. A549 cells were plated to six-well plates and treat with exosomes when they were attached. Then, monolayer cells were manually wounded by scraping the cells with a pipette tip and washed with PBS before the culture medium was replaced. After 24 h of incubation, progression of migration was observed and photographed by using an inverted microscope (Carl Zeiss, Germany).

Statistical analyses

Graphpad Prism version 5.01 was used for statistical analyses. Data are expressed as means ± standard deviations (SD). Statistical significance was determined using one-way

ANOVA or unpaired two-tailed Student's t-test. $P < 0.05$ was considered statistically significant.

References

1. Vella LJ, Scicluna BJ, Cheng L, Bawden EG, Masters CL, Ang CS, et al. A rigorous method to enrich for exosomes from brain tissue. Journal of extracellular vesicles. 2017; 6: 1348885.
2. Lu Y, Yu T, Liang H, Wang J, Xie J, Shao J, et al. Nitric oxide inhibits hetero-adhesion of cancer cells to endothelial cells: restraining circulating tumor cells from initiating metastatic cascade. Scientific reports. 2014; 4: 4344.
3. Lian S, Lu Y, Cheng Y, Yu T, Xie X, Liang H, et al. S-nitrosocaptopril interrupts adhesion of cancer cells to vascular endothelium by suppressing cell adhesion molecules via inhibition of the NF-small ka, CyrillicB and JAK/STAT signal pathways in endothelial cells. European journal of pharmacology. 2016; 791: 62-71.
4. Wen M, Shen Y, Shi S, Tang T. miREvo: an integrative microRNA evolutionary analysis platform for next-generation sequencing experiments. BMC bioinformatics. 2012; 13: 140.
5. Friedlander MR, Mackowiak SD, Li N, Chen W, Rajewsky N. miRDeep2 accurately identifies known and hundreds of novel microRNA genes in seven animal clades. Nucleic acids research. 2012; 40: 37-52.
6. Young MD, Wakefield MJ, Smyth GK, Oshlack A. Gene ontology analysis for RNA-seq: accounting for selection bias. Genome biology. 2010; 11: R14.
7. Lu Y, Lian S, Ye Y, Yu T, Liang H, Cheng Y, et al. S-Nitrosocaptopril prevents cancer metastasis in vivo by creating the hostile bloodstream microenvironment against circulating tumor cells. Pharmacological research. 2019; 139: 535-49.
8. Hirata H, Hinoda Y, Shahryari V, Deng G, Tanaka Y, Tabatabai ZL, et al. Genistein downregulates onco-miR-1260b and upregulates sFRP1 and Smad4 via demethylation and histone modification in prostate cancer cells. British journal of cancer. 2014; 110: 1645-54.

Supplementary figures

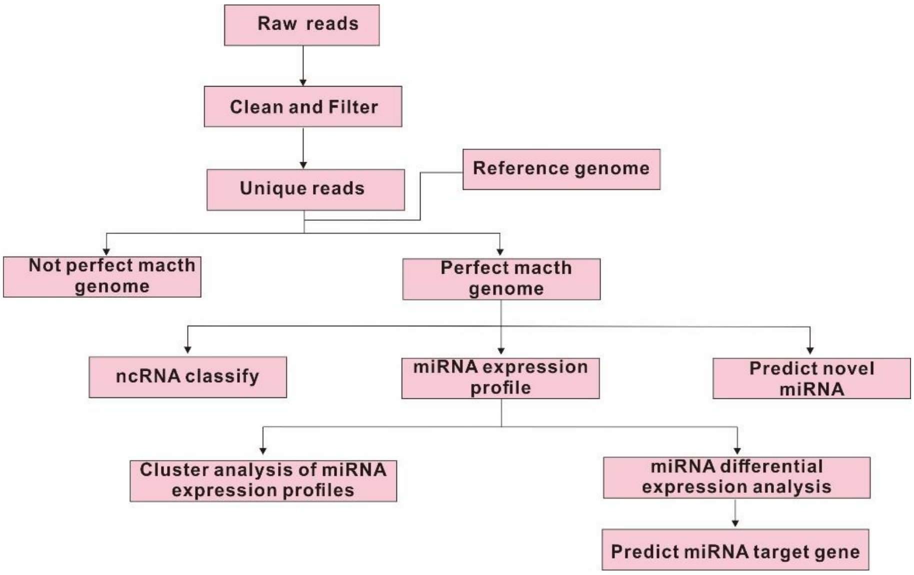

Figure S1. Schematic diagram of the miRNA analysis process.

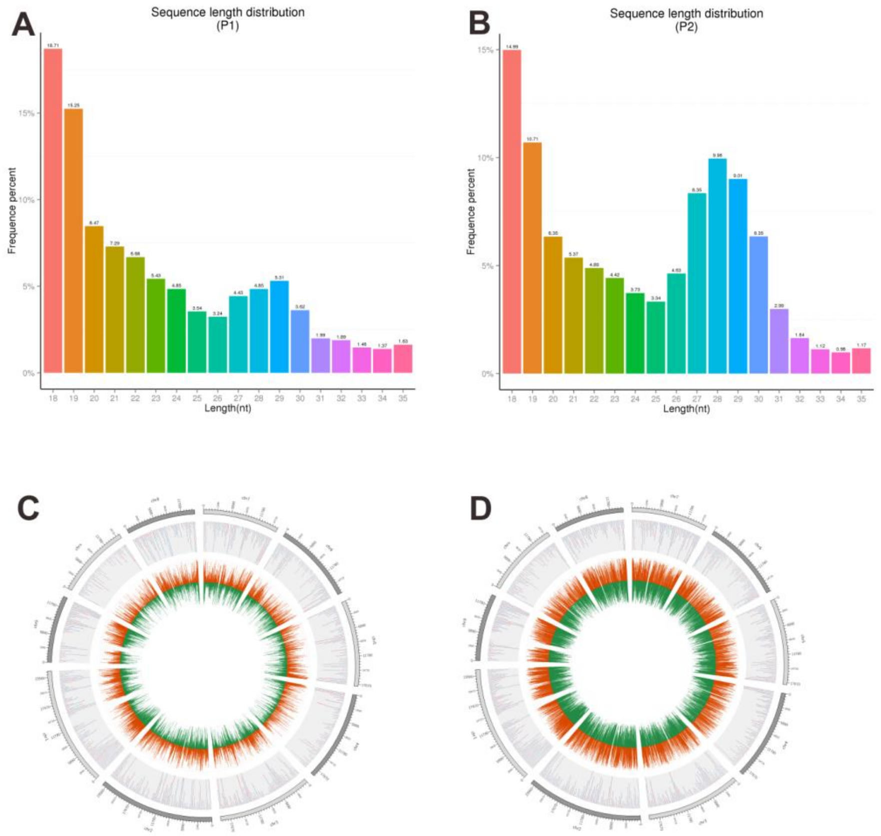

Figure S2. A-B,The sequence length distribution of sRNA in E-exosome(**A**) and A-exosome(**B**). **C-D,** Circos mapping show the distribution of reads on each chromosome. The outermost is the selection of displayed chromosomes. The grey background area in the

middle is the distributions of extracted 10000 reads, the red mapping to the positive chain, and the blue mapping to the negative chain. The innermost circles are all reads on the chromosome, orange is positively linked to coverage, and green is negative chain coverage distribution.

Supplementary tables

Table S1. List of RNA sequence filtering results

Sample	total reads	N%>10%	low quality	5 adapter contamine	3 adapter null or insert null	with ploy A/T/G/C	clean reads
E-exosome	22000000 (100.00%)	2696 (0.01%)	0(0.00%)	7075(0.03%)	1665472 (7.57%)	235782(1.07%)	20088975 (91.31%)
A-exosome	22000000 (100.00%)	2859 (0.01%)	0(0.00%)	6580(0.03%)	2089114 (9.50%)	251176(1.14%)	19650271 (89.32%)

Table S2. Comparison of sRNA with reference genome

Sample	Total sRNA	Mapped sRNA	+ Mapped sRNA	- Mapped sRNA
E-exosome	13029934(100.00%)	1873283(14.38%)	736317(5.65%)	1136966(8.73%)
A-exosome	10548350(100.00%)	1911922(18.13%)	784182(7.43%)	1127740(10.69%)

Table S3. Differential analysis results of miRNA expression

sRNA	E-exosome	A-exosome	log2.Fold_change.	p.value	Signature
novel_10	0	41403.33591	-16.337	0	TRUE
novel_113	0	17744.28682	-15.115	0	TRUE
novel_117	0	13012.477	-14.668	0	TRUE
novel_118	6699.30723	0	13.71	0	TRUE
novel_12	0	14195.42945	-14.793	0	TRUE
novel_125	64121.94063	42586.28836	0.59043	0	FALSE
novel_128	44024.01894	0	16.426	0	TRUE
novel_129	0	31939.71627	-15.963	0	TRUE
novel_134	3828.17556	0	12.902	0	TRUE
novel_137	1914.08778	0	11.902	0	TRUE
novel_139	8134.873066	0	13.99	0	TRUE
novel_140	0	33122.66873	-16.016	0	TRUE
novel_144	4306.697505	0	13.072	0	TRUE
novel_15	21054.96558	0	15.362	0	TRUE
novel_153	0	28390.85891	-15.793	0	TRUE
novel_163	11006.00474	0	14.426	0	TRUE
novel_168	0	4731.809818	-13.208	0	TRUE

novel_172	6699.30723	0	13.71	0	TRUE
novel_177	0	18927.23927	-15.208	0	TRUE
novel_181	0	22476.09664	-15.456	0	TRUE
novel_184	0	47318.09818	-16.53	0	TRUE
novel_190	10527.48279	0	14.362	0	TRUE
novel_192	18662.35586	0	15.188	0	TRUE
novel_194	0	28390.85891	-15.793	0	TRUE
novel_197	0	30756.76382	-15.909	0	TRUE
novel_199	0	7097.714727	-13.793	0	TRUE
novel_200	5742.26334	0	13.487	0	TRUE
novel_202	0	39037.431	-16.253	0	TRUE
novel_205	3349.653615	0	12.71	0	TRUE
novel_208	1914.08778	0	11.902	0	TRUE
novel_210	0	43769.24082	-16.418	0	TRUE
novel_222	0	20110.19173	-15.296	0	TRUE
novel_224	4785.21945	0	13.224	0	TRUE
novel_226	0	33122.66873	-16.016	0	TRUE
novel_227	0	30756.76382	-15.909	0	TRUE
novel_234	13877.13641	0	14.76	0	TRUE
novel_236	0	23659.04909	-15.53	0	TRUE
novel_245	26318.70698	0	15.684	0	TRUE
novel_247	0	36671.52609	-16.162	0	TRUE
novel_249	1914.08778	0	11.902	0	TRUE
novel_252	18183.83391	0	15.15	0	TRUE
novel_258	3349.653615	0	12.71	0	TRUE
novel_259	0	9463.619636	-14.208	0	TRUE
novel_261	3349.653615	0	12.71	0	TRUE
novel_262	0	7097.714727	-13.793	0	TRUE
novel_264	0	33122.66873	-16.016	0	TRUE
novel_267	6220.785285	0	13.603	0	TRUE
novel_269	0	16561.33436	-15.016	0	TRUE
novel_27	3828.17556	0	12.902	0	TRUE
novel_271	4785.21945	0	13.224	0	TRUE
novel_284	0	24842.00154	-15.6	0	TRUE
novel_308	0	20110.19173	-15.296	0	TRUE
novel_313	0	26024.954	-15.668	0	TRUE
novel_324	0	37854.47854	-16.208	0	TRUE
novel_329	20097.92169	0	15.295	0	TRUE
novel_33	0	30756.76382	-15.909	0	TRUE
novel_334	4785.21945	0	13.224	0	TRUE
novel_339	33975.0581	0	16.052	0	TRUE
novel_34	0	41403.33591	-16.337	0	TRUE
novel_343	0	18927.23927	-15.208	0	TRUE
novel_349	3349.653615	3548.857364	-0.083343	0	FALSE

novel_351	0	46135.14573	-16.494	0	TRUE
novel_357	0	3548.857364	-12.793	0	TRUE
novel_359	0	28390.85891	-15.793	0	TRUE
novel_365	6220.785285	0	13.603	0	TRUE
novel_373	2392.609725	0	12.224	0	TRUE
novel_378	2871.13167	0	12.487	0	TRUE
novel_383	19140.8778	0	15.224	0	TRUE
novel_394	0	67428.28991	-17.041	0	TRUE
novel_401	8134.873066	0	13.99	0	TRUE
novel_402	0	8280.667182	-14.016	0	TRUE
novel_408	0	17744.28682	-15.115	0	TRUE
novel_409	19619.39975	0	15.26	0	TRUE
novel_413	7177.829176	0	13.809	0	TRUE
novel_418	5742.26334	0	13.487	0	TRUE
novel_426	5742.26334	0	13.487	0	TRUE
novel_439	4306.697505	0	13.072	0	TRUE
novel_448	6220.785285	0	13.603	0	TRUE
novel_449	0	27207.90645	-15.732	0	TRUE
novel_457	0	20110.19173	-15.296	0	TRUE
novel_464	0	16561.33436	-15.016	0	TRUE
novel_467	0	17744.28682	-15.115	0	TRUE
novel_470	0	15378.38191	-14.909	0	TRUE
novel_474	0	18927.23927	-15.208	0	TRUE
novel_477	8613.395011	0	14.072	0	TRUE
novel_481	0	15378.38191	-14.909	0	TRUE
novel_487	0	14195.42945	-14.793	0	TRUE
novel_491	4785.21945	0	13.224	0	TRUE
novel_497	4785.21945	0	13.224	0	TRUE
novel_498	0	40220.38345	-16.296	0	TRUE
novel_502	7656.351121	0	13.902	0	TRUE
novel_512	0	57964.67027	-16.823	0	TRUE
novel_515	4785.21945	0	13.224	0	TRUE
novel_516	3349.653615	29573.81136	-3.1422	0	TRUE
novel_526	0	46135.14573	-16.494	0	TRUE
novel_53	3349.653615	0	12.71	0	TRUE
novel_538	0	14195.42945	-14.793	0	TRUE
novel_539	8613.395011	0	14.072	0	TRUE
novel_540	0	35488.57364	-16.115	0	TRUE
novel_544	0	18927.23927	-15.208	0	TRUE
novel_551	5263.741395	0	13.362	0	TRUE
novel_559	3828.17556	0	12.902	0	TRUE
novel_564	0	40220.38345	-16.296	0	TRUE
novel_57	0	17744.28682	-15.115	0	TRUE
novel_582	0	7097.714727	-13.793	0	TRUE

novel_585	0	8280.667182	-14.016	0	TRUE
novel_59	5263.741395	0	13.362	0	TRUE
novel_6	0	26024.954	-15.668	0	TRUE
novel_64	0	29573.81136	-15.852	0	TRUE
novel_67	21533.48753	0	15.394	0	TRUE
novel_7	1914.08778	0	11.902	0	TRUE
novel_73	2392.609725	0	12.224	0	TRUE
novel_78	11963.04863	0	14.546	0	TRUE
novel_82	0	35488.57364	-16.115	0	TRUE
novel_83	11963.04863	0	14.546	0	TRUE
novel_84	0	16561.33436	-15.016	0	TRUE
novel_87	5742.26334	0	13.487	0	TRUE
novel_88	18183.83391	0	15.15	0	TRUE
novel_89	6220.785285	0	13.603	0	TRUE
novel_157	1435.565835	0	11.487	########	TRUE
novel_253	1435.565835	0	11.487	########	TRUE
novel_434	1435.565835	0	11.487	########	TRUE
novel_86	1435.565835	0	11.487	########	TRUE
novel_119	0	2365.904909	-12.208	########	TRUE
novel_74	957.0438901	0	10.902	########	TRUE
hsa-miR-1260b	0	1182.952455	-11.208	########	TRUE
novel_100	0	1182.952455	-11.208	########	TRUE
novel_120	0	1182.952455	-11.208	########	TRUE
novel_196	0	1182.952455	-11.208	########	TRUE
novel_217	0	1182.952455	-11.208	########	TRUE
novel_341	0	1182.952455	-11.208	########	TRUE
novel_354	0	1182.952455	-11.208	########	TRUE
novel_360	0	1182.952455	-11.208	########	TRUE
novel_392	0	1182.952455	-11.208	########	TRUE
novel_395	0	1182.952455	-11.208	########	TRUE
novel_420	0	1182.952455	-11.208	########	TRUE
novel_431	0	1182.952455	-11.208	########	TRUE
novel_450	0	1182.952455	-11.208	########	TRUE
novel_455	0	1182.952455	-11.208	########	TRUE
novel_458	0	1182.952455	-11.208	########	TRUE
novel_488	0	1182.952455	-11.208	########	TRUE
novel_510	0	1182.952455	-11.208	########	TRUE
novel_558	0	1182.952455	-11.208	########	TRUE
novel_567	0	1182.952455	-11.208	########	TRUE
novel_92	0	1182.952455	-11.208	########	TRUE
hsa-miR-718	478.521945	0	9.9024	########	TRUE
novel_101	478.521945	0	9.9024	########	TRUE
novel_127	478.521945	0	9.9024	########	TRUE
novel_14	478.521945	0	9.9024	########	TRUE

novel_170	478.521945	0	9.9024	########	TRUE
novel_211	478.521945	0	9.9024	########	TRUE
novel_220	478.521945	0	9.9024	########	TRUE
novel_233	478.521945	0	9.9024	########	TRUE
novel_294	478.521945	0	9.9024	########	TRUE
novel_311	478.521945	0	9.9024	########	TRUE
novel_325	478.521945	0	9.9024	########	TRUE
novel_337	478.521945	0	9.9024	########	TRUE
novel_352	478.521945	0	9.9024	########	TRUE
novel_376	478.521945	0	9.9024	########	TRUE
novel_382	478.521945	0	9.9024	########	TRUE
novel_441	478.521945	0	9.9024	########	TRUE
novel_446	478.521945	0	9.9024	########	TRUE
novel_453	478.521945	0	9.9024	########	TRUE
novel_454	478.521945	0	9.9024	########	TRUE
novel_466	478.521945	0	9.9024	########	TRUE
novel_543	478.521945	0	9.9024	########	TRUE
novel_56	478.521945	0	9.9024	########	TRUE
novel_560	478.521945	0	9.9024	########	TRUE
novel_572	478.521945	0	9.9024	########	TRUE
novel_61	478.521945	0	9.9024	########	TRUE
novel_79	478.521945	0	9.9024	########	TRUE

Table S4. Novel miRNA sequence list

**sRNA**	**Sequence** (5'-3')
>novel_10	ucgcgggcgcggggcaca
>novel_100	uccgcgggggcggguccccc
>novel_101	cccaggagggggcggcuc
>novel_113	cccccuggggggaaggcgg
>novel_117	cccgggccgggggcugcc
>novel_118	ccggcgggcgcggggcga
>novel_119	gccgggggcggcgggggac
>novel_12	gcccggcgggcggggcuc
>novel_120	cacaggguugguggaagg
>novel_125	gugcagcgcacgugcucc
>novel_127	cugcagcgcccggcgcaa
>novel_134	gcccggcuggggcggugg
>novel_137	cccaggcuggggcggggc
>novel_139	cuccaggggaaggggcccc
>novel_14	ccguaccccggggccgcuc
>novel_140	caggccggcggggacaac
>novel_144	cccgccgcgggggugggg

>novel_15	acccugggucggggcagc
>novel_153	cgccgggcggcggggcug
>novel_157	cagcgagggcugggcugac
>novel_163	cacagaggggugguggc
>novel_168	ccagggcggcggggcgcg
>novel_170	cucagagagaggggcaagu
>novel_172	ccggggcccggguugggg
>novel_177	gccaggcccuggcggggauu
>novel_181	uuccagggcugggucucug
>novel_184	cuucaccgccugggcuuc
>novel_190	cccugaagagggggguagg
>novel_192	caggagggagggaacugac
>novel_194	ccgcagcgggcgggggca
>novel_196	cgccgccggcggggccac
>novel_197	cuucagagaggggcccuucc
>novel_199	ccccagggccccgcaccc
>novel_200	cccagaagagggggccuc
>novel_202	acucgagccgggcgcggu
>novel_205	cccgcggguggggugguu
>novel_208	cccagggugggggcucu
>novel_210	guguagggcugggugcgg
>novel_211	cacggggaggcccgggaac
>novel_217	aagcgcagggcccggagc
>novel_220	aacaagcacccggccuccc
>novel_222	ugccaagggcagggcagg
>novel_224	ccuaggguggggggguggg
>novel_226	ccgccgcggcggggggug
>novel_227	ccucaggaaggggauucc
>novel_233	ccagagggcugcgucucac
>novel_234	uagcagggcugggugccc
>novel_236	gguuucccggcggcguccc
>novel_245	ccccagagaggggcccgucuc
>novel_247	cccugggucggggcuguc
>novel_249	gcccgggcuggagaggugg
>novel_252	cuccaccccgcggacguc
>novel_253	agacaggggcccgggacac
>novel_258	cuccaggcaggaggaugu
>novel_259	cgcagggagcgggggugc
>novel_261	aaccagggcuggguguga
>novel_262	cgccaagggcaggggagc
>novel_264	gacaaagggcagggacuc
>novel_267	agcuggaggcugaggguag
>novel_269	gcguagggcuggguaggg
>novel_27	cagagcgagggggguccg

>novel_271	ccuuggggaagggggguca
>novel_284	cagcaggggcuggggcua
>novel_294	caaggagagagggggcuc
>novel_308	acagaagggcuggggagg
>novel_311	cccggagaggggccuugg
>novel_313	gcccagggcuggguagaggc
>novel_324	ccgagggcgcggggaugc
>novel_325	aaagagggcuggguccugc
>novel_329	ccuagagagaggggagac
>novel_33	ccggggcuggggcggucc
>novel_334	cacucccggugagcucuggg
>novel_337	ucaagagagaggggcuug
>novel_339	ggccgggcgugguggagac
>novel_34	ggccggccgccggacccc
>novel_341	agccaagggacggggacc
>novel_343	cagccuggcuggagcuug
>novel_349	gcgccgggcgcggggagac
>novel_351	cccgccgcgggggcucuc
>novel_352	ccggcggcccggacccuc
>novel_354	cugcagggcuggguugca
>novel_357	ccaguuucuggcaccugac
>novel_359	ccagagggcuggguc aug
>novel_360	acaagagggaggggucc c
>novel_365	ccagaagggcaggggagu
>novel_373	cucagagagaggggucag
>novel_376	ccuugaggagggggaggag
>novel_378	ccucaagaggggggagaua
>novel_382	ccagccccggccggucca
>novel_383	cccugggugggggcucc
>novel_392	ucccuuugggaggcaggg
>novel_394	ccagagggcugggugagg
>novel_395	acaaagggcuggguacaa
>novel_401	cccagggugggggaugg
>novel_402	ugccaagggcagggaugc
>novel_408	gagcagggggacggggac
>novel_409	caucgggcuggagaggguc
>novel_413	cccagccuggggaggccc
>novel_418	ccuggagagaggggcucug
>novel_420	ccagggggcuggguuugg
>novel_426	cccuggggagggggaugu
>novel_431	aaagagagggaaggcaca
>novel_434	caggggucccggguucugg
>novel_439	cccugaagagggggauuuuc
>novel_441	ggcccugcccucugggcac

>novel_446	augcagggcugggucugc
>novel_448	ccaggaccgccugggggua
>novel_449	cccugaagagggggaugu
>novel_450	cacaaagggauggggaug
>novel_453	ggccauguagaggcaggg
>novel_454	gcagaggcugggcagugcc
>novel_455	gcccaagggacgggaggg
>novel_457	aaagaggcgggagauuca
>novel_458	cacggucacaguggcuccc
>novel_464	gaguggaggggggaaagag
>novel_466	cauacggggcugggccuc
>novel_467	cuaugggucgggcgcggug
>novel_470	cuccaggcugaggccuca
>novel_474	aaaggaggcugaggcuua
>novel_477	ccacaagggcagggcuca
>novel_481	gugggggcuggagagaggaa
>novel_487	caguagggcugggugcag
>novel_488	ucuggggucuguagcaaac
>novel_491	cccagggcuggagagggag
>novel_497	cccggggugggggugcg
>novel_498	uuccaagggcagggcacc
>novel_502	cccuggggugggggcagcc
>novel_510	gacucgggucgggguccc
>novel_512	cccaaggccuacccacac
>novel_515	cucaaagagggggagggg
>novel_516	ccacgaagagggggagug
>novel_526	gccagcguugguggguga
>novel_53	gccugggagggcgcgggu
>novel_538	cagcgcguuuguggguag
>novel_539	cccaagagaggggaggaa
>novel_540	aacuaagggcaggggaug
>novel_543	guuugggcuagagaucug
>novel_544	aauugucuggagaagggca
>novel_551	ccucagagaggggcugcc
>novel_558	cugcagguuggggauugc
>novel_559	cagaaagggcaggggcag
>novel_56	cccgaggcgggggaaccg
>novel_560	accaggagauggggugaggg
>novel_564	cagggaccagccuggagac
>novel_567	aacagcgugggugggaua
>novel_57	ccgcgccggcgggaggagg
>novel_572	gcuuagggcugggugccg
>novel_582	ccgcggagcgggagcgcg
>novel_585	gcagggcuggggcggagg

>novel_59	cgcgggcggggcgggugg
>novel_6	cgccggcggcggggcagc
>novel_61	cccgggcggcggcgacac
>novel_64	agcgcccgcgggggucgu
>novel_67	ugccgggugggcggcccc
>novel_7	caccagcgcccggcgcgu
>novel_73	cgccgagggggcggggga
>novel_74	cgcaccccagccucccuc
>novel_78	cgaggaagcccggcugcc
>novel_79	uaagagcgcccgggcuucc
>novel_82	ggagaaggggagggggcc
>novel_83	ccguggggguggggagc
>novel_84	gcccggacgggcgaguca
>novel_86	cgcgcccggcggcggcug
>novel_87	ccggacccggcggcgaggg
>novel_88	ccccagcgggcgggcggcagc
>novel_89	cuccgcggcgggggcgcc
>novel_92	cccgggagggggcgcgcc

Table S5. Target gene prediction results

miRNA	target_mRNA	target_gene
hsa-miR-1260b	ENST00000000412	ENSG00000003056
hsa-miR-1260b	ENST00000000442	ENSG00000173153
hsa-miR-1260b	ENST00000002829	ENSG00000001617
hsa-miR-1260b	ENST00000003583	ENSG00000001460
hsa-miR-1260b	ENST00000003912	ENSG00000001461
hsa-miR-1260b	ENST00000004531	ENSG00000003989
hsa-miR-1260b	ENST00000005178	ENSG00000004799
hsa-miR-1260b	ENST00000006015	ENSG00000005073
hsa-miR-1260b	ENST00000006053	ENSG00000006210
hsa-miR-1260b	ENST00000006275	ENSG00000007255
hsa-miR-1260b	ENST00000006777	ENSG00000005486
hsa-miR-1260b	ENST00000007510	ENSG00000004777
hsa-miR-1260b	ENST00000007633	ENSG00000006534
hsa-miR-1260b	ENST00000009180	ENSG00000010278
